# Case report: Anxiety and depression as initial symptoms in a patient with acute hypoxia and patent foramen ovale

**DOI:** 10.3389/fpsyt.2023.1229995

**Published:** 2023-08-22

**Authors:** Xiaoyan Zhai, Ronghong Jiao, Aihua Ni, Xueyi Wang

**Affiliations:** ^1^Department of Clinical Psychology, Hebei General Hospital, Shijiazhuang, China; ^2^Department of Ultrasound, Hebei General Hospital, Shijiazhuang, Hebei, China; ^3^Psychiatric Department of The First Hospital of Hebei Medical University, Institute of Mental Health of Hebei Medical University, Shijiazhuang, Hebei, China

**Keywords:** anxiety, depression, hypoxia, right-to-left shunt, patent foramen ovale, agitated

## Abstract

The prevalence of patent foramen ovale (PFO) is 15–35% among adults. The role of right-to-left shunting through the PFO, anxiety, depression, and hypoxemia in the systemic circulation remains poorly understood. Herein, we present the case of a 52-year-old woman with no heart or lung disease, who was admitted due to anxiety for 5 months and had symptom exacerbation with dizziness for 4 days and presented with cyanosis. She was noted to have acute hypoxemia, with an oxygen saturation of 94.48% on room air, and arterial blood gas showed an oxygen tension of 65.64 mmHg. Agitated saline contrast echocardiography showed right-to-left shunting due to PFO. Arteriovenous fistula, pneumonia, pulmonary embolism, pulmonary hypertension, congestion peripheral cyanosis, ischemic peripheral cyanosis, and methemoglobin were excluded. Additionally, the patient improved by taking Paroxetine, Oxazepam, and Olanzapine. Her oxygen tension returned to 90.42 mmHg, and her symptoms resolved. In the case of severe anxiety and depression, right-to-left shunting through the PFO may cause acute systemic hypoxemia *via* a flow-driven mechanism, occasionally manifesting as cyanosis. When anxiety improved, hypoxia also improved. Thus, the treatment of anxiety and depression seems effective in improving hypoxemia. Notably, this is a rare report, and we hope to draw the attention of psychosomatic specialists, psychiatrists, and clinicians to seek the relationship between anxiety appearing as acute stress and PFO. This may be a new therapeutic method for treating severe anxiety disorder.

## 1. Introduction

Anxiety disorders are the most common type of mental illness in China (1). High prevalence, comorbidity, and chronicity led the WHO to rank anxiety disorders as the ninth biggest health-related cause of disability (2). Notably, environmental factors (3) and genetic factors (4) influence the risk of anxiety disorders. Many brain imaging studies have also shown structural alterations in anxiety disorders within the medial temporal, prefrontal cortex, and cingulate regions (5, 6). However, we are uncertain of how it happened and whether there were any substances passed *via* certain channels, causing anxiety disorder.

Usually, the venous system’s thrombi, air, fat, and other emboli cannot pass through the pulmonary circulation. It will not enter the systemic circulation to cause embolism unless abnormal channels in the heart (such as patent foramen ovale) exist. Patent foramen ovale (PFO) is a common defect of the interatrial septum, with an incidence of 15–35% in the adult population (7). Most people with PFO are completely asymptomatic throughout life (8). The left atrial pressure is usually higher than the right, preventing blood flow against the gradient (9). However, when a patient suddenly coughs, takes a deep breath, or performs a Valsalva maneuver, the right atrial pressure is higher than the left atrial pressure, and a right-to-left shunt occurs at the atrial level. Furthermore, the embolus can enter the left heart directly without passing through the lungs, thus leading to unknown causes of embolism, stroke, and other diseases. In some subjects, it can be associated with stroke or transient ischemic attacks due to paradoxical embolization (10), migraine with aura (11), decompression sickness (12, 13), or hypoxemic medical conditions^[9]^because of its right-to-left shunting. Furthermore, anatomic and physiologic changes may increase right-to-left shunting, resulting in more severe hypoxemia (14). Transesophageal echocardiography with the addition of agitated saline contrast and appropriately performed provocative maneuvers is considered the gold-standard imaging modality for identifying an intracardiac shunt, especially patent foramen ovale (15).

Herein, we report a unique and interesting case of acute systemic hypoxemia caused by right-to-left shunting through the PFO in a patient with severe anxiety and depression.

## 2. Case report

A 52-year-old woman was admitted due to anxiety and depression for 5 months and symptom exacerbation with dizziness for 4 days (Table 1). She felt high work stress 5 months previously, then the symptoms occurred. Symptoms reported included upset, restlessness, nervousness, worry, apprehension, being easily frightened, gloom, loss of energy, decreased interest, insomnia, tension headache, shortness of breath, dizziness, nausea, and poor appetite. Thus, it was diagnosed as anxiety and depression. The patient felt better when administered venlafaxine 225 mg once a day. However, the abrupt discontinuation of the drug happened 4 days previously, and the patient subsequently experienced dizziness, nausea, vomiting, sweating, nervousness, fear, increased anxiety, and progressive shortness of breath. She had no previous history of psychiatry, hypertension, diabetes mellitus, or coronary artery disease. No smoking or drinking habits were reported, and no family history of mental illness was reported. Furthermore, there were no positive signs on the cardiopulmonary, abdominal, and nervous systems. Notably, the mental health examination demonstrated that the patient was nervous, her eyebrows constricted, but there was no history of hallucinations or delusions.

**Table 1:** The patient’s disease course and timeline.

Date	Treatment	Arterial blood gas analysis			HAMA	D-dimer quantitation (mg/L)	Clinical manifestations
		PaO_2_ (mmHg)	SaO_2_ (%)	PaCO_2_ (mmHg)			
Day 1 (2021.9.3)	before treatment	65.64	94.48	44.22	HAMA:26 HAMD_24_:31	0.6	anxiety, depression, insomnia, shortness of breath, nausea, dizziness, cyanosis, etc.
Day 3 (2021.9.5)	Oxygen, Paroxetine, oxazepam, olanzapine	89.47 (Stop oxygen inhaled)	98.29	48.06	–	normal	insomnia, shortness of breath, nausea, dizziness, cyanosis disappeared. Upset, restlessness, nervousness, squeezed headache improved
Day 11 (2021.9.13)	Paroxetine, oxazepam, olanzapine	90.42	97.88	48.42	HAMA:1 HAMD_24_:1	–	anxiety, depression, and physical symptoms improved

**Figure 1:**
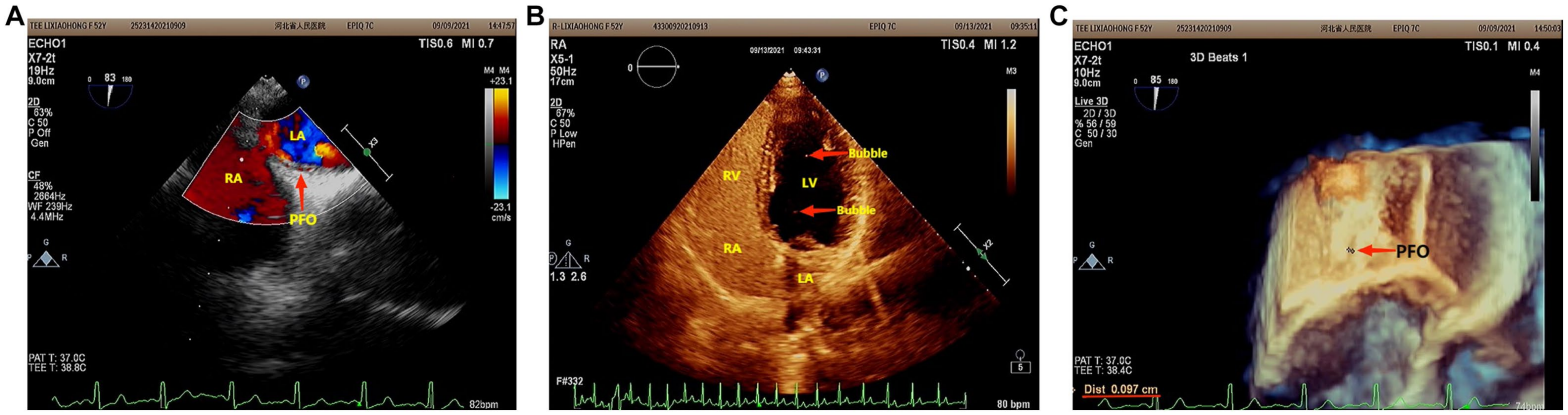
**(A)** Agitated saline contrast echocardiography showed that the maximum number of bubbles in a single frame was less than 10 bubbles (RLS 1) in three cardiac cycles after the Valsalva maneuver. **(B)** Two-dimensional echocardiography of the transesophageal heart showed that there was a 0.9 mm wide fissure echo in the middle ovoid fossa of the atrial septum. **(C)** Three-dimensional echocardiography of the Transesophageal Heart: After the Valsalva maneuver, the width of the ovoid fossa of the atrial septum was approximately 0.9 mm (star-like shunt was occasionally seen).

**Figure 2:**
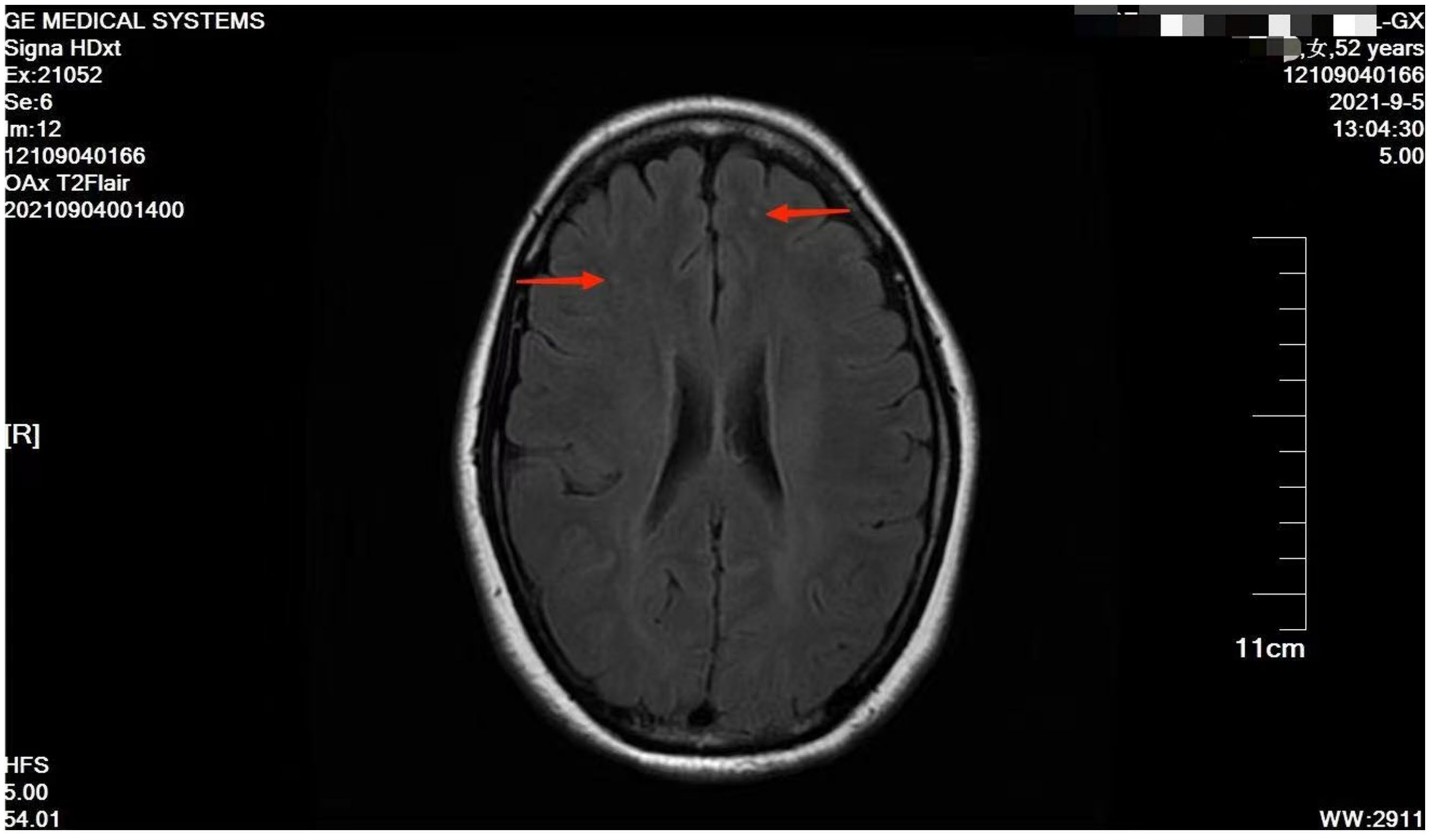
MRI T2 flare showed bilateral frontal lobe chronic small ischemic focus.

However, she presented with cyanosis and was found to be hypoxic, with an oxygen saturation of 94.48% on room air. The arterial blood gas showed an oxygen tension of 65.64 mmHg. Routine blood tests, biochemistry, ESR, thyroid function, cortisol, female tumor examination, chest CT, ultrasound of deep and intermuscular veins of both lower limbs, and Holter monitor were normal. D-dimer quantitation was 0.6 mg/L (0–0.55), and prolactin was reported as 42.86 ng/mL. Echocardiography: mild aortic, mitral, and tricuspid regurgitation. Agitated saline contrast echocardiography was positive (RLS grade 1; Figure 1). Transesophageal echocardiography showed a fossa ovoid fissure, approximately 0.9 mm wide (occasionally stellate shunt; Figures 1B,C). Additionally, an MRI image showed bilateral frontal lobe chronic small ischemic focus (Figure 2), and MRA results were normal. EPQ: P 62, N 46, E 64, L 44. SCL-90: the total score was 205, the number of positive symptoms was 65, anxiety was 2.9, phobia was 2.67, somatization was 2.33, psychosis was 1.9, obsessive–compulsive was 2.4, depression was 2.38, and other symptoms were 2.57. HAMA:26, HAMD_24_:31. These indicated that the patient had severe anxiety and depression accompanied by hypoxemia. Medical history, along with symptoms, signs, and examination showed that hypoxemia and cyanosis were not caused by arteriovenous fistula, pneumonia, pulmonary embolism, thrombophlebitis, pericarditis, right heart failure, severe shock, Raynaud’s disease, occlusive vasculitis, methemoglobin due to nitrite, sulfur-containing drug intake, or ampicillin poisoning. Acute nausea and vomiting may have been related to venlafaxine’s sudden withdrawal. Therefore, the patient was diagnosed with anxiety disorder, depressive episodes, and hypoxemia (ICD-10). As a result, she was prescribed paroxetine and oxazepam to improve her anxiety, depression, and insomnia. Olanzapine can improve physical symptoms such as nausea and vomiting and help to improve insomnia, anxiety, and depression (16). Meanwhile, oxygen inhalation was carried out. When other structural abnormalities or pathological changes in the lungs and heart were ruled out, we found the patient suffered from PFO (Figure 1). Interestingly, only 2 days later, when insomnia, shortness of breath, nausea, dizziness, and anxiety symptoms improved, PaO_2_ increased to 89.47 mmHg. Furthermore, cyanosis disappeared, and D-dimer returned to normal (as is widely understood, a hypercoagulable state is associated with severe anxiety (17)). Thus, we stopped oxygen inhalation, and PaO_2_ did not decrease (Table 1). Based on the above results, we speculated that physical stress (nausea, shortness of breath, etc.) and emotional stress are major stressors for patients, leading to cyanosis and acute, transient hypoxia due to increased right-to-left shunt. When anxiety, depression, nausea, and other stressors were relieved, the right-to-left shunt may have been small, and acute hypoxia was eventually improved. Six months later, the patient insisted on low oral doses of paroxetine and olanzapine, felt stable, and no longer appeared to present with cyanosis or hypoxia.

## 3. Discussion

Our study reports a rare and interesting case of PFO-associated acute systemic hypoxemia presenting with cyanosis induced by severe anxiety and depression. When people are exposed to stressors, such as painful stimulation, either physically or from the environment, many measurement studies report increased stress responses in people with anxiety disorders versus controls (3). As in this case report, sudden withdrawal caused nausea, an acute stress event, and increased anxiety. Thus, the patient presented with increased anxiety, shortness of breath, cyanosis, and so on. When anxiety improved, anoxia also improved quickly. Notably, this may be associated with a flow-driven or pressure-driven mechanism (14). This case highlights an important point: right-to-left shunting through a PFO could cause severe systemic hypoxemia and cyanosis. It may also be caused by severe anxiety, and depression appears as acute stress. Thus, these triggered us to hypothesize that stress may cause or aggravate the right-to-left shunt through a PFO. This case also highlights the importance of considering underlying medical conditions when evaluating patients with psychiatric symptoms. Thus, clinicians should maintain a high index of suspicion for underlying medical conditions. Notably, this case report underscores the significance of a comprehensive evaluation of patients with anxiety and depression. Furthermore, clinicians should consider the possibility of underlying medical conditions, including cardiovascular abnormalities, even in the absence of typical symptoms. Therefore, early recognition and appropriate management can likely improve outcomes and resolve psychiatric symptoms.

Patent foramen ovale is a remnant of fetal circulation (7). Hemodynamic or anatomic changes can cause right-to-left shunting through the PFO *via* pressure-driven or flow-driven mechanisms (18). Additionally, PFO-associated hypoxemia occurs when deoxygenated venous blood from the right atrium enters and mixes with oxygenated arterial blood in the left atrium (19). This phenomenon is commonly associated with pulmonary pathologies such as chronic obstructive pulmonary disease, obstructive sleep apnea (14), and platypnea-orthodeoxia syndrome (20). Notably, patients with a right-to-left shunt may have profound hypoxemia that is out of proportion to underlying primary lung disease, even with no pneumonia, pulmonary embolism, pulmonary hypertension, or arteriovenous fistula (18, 21). We infer that acute stress-induced dysautonomia may also play an important role. Acute stress due to sudden withdrawal and aggravation of anxiety leads to autonomic dysfunction. Consequently, dysautonomia causes changes in pressure-driven or flow-driven mechanisms, exacerbating the right-to-left shunt. Paroxetine, belonging to the selective serotonin reuptake inhibitors, is used to improve anxiety and depression (22). Oxazepam is a short-acting benzodiazepine anxiolytic used to treat alcohol withdrawal and manage anxiety disorders and tension (23). Olanzapine belongs to the second-generation antipsychotics and affects the serotonin, dopamine, adrenaline, histamine, and muscarinic systems. Apart from dealing with delirium, it includes the management of nausea, vomiting, and loss of appetite (16). When these pharmacotherapies worked, there was an overall improvement in stress, dysautonomia, flow-driven or pressure-driven mechanisms due to right-to-left shunt, and hypoxia. Furthermore, the patient no longer needed supplementary oxygen.

One theory suggests that the brain-heart axis connects frontal and limbic brain regions to the brainstem and periphery *via* the autonomic nervous system (24). The density of the sympathetic nervous system varies from the atria to the ventricles and from the bottom of the heart to the apex. The sympathetic nervous system of the atria was the densest (25, 26). Thus, we inferred that acute stress leads to dysautonomia and that the atrial autonomic nerves are densely packed, making it possible for the right-to-left shunt to worsen. Notably, when physical and/or psychological stress improved, autonomic dysfunction was improved, right-to-left shunt was controlled, and hypoxia was improved. Similarly, Takotsubo syndrome, also caused by acute stress, mediates the stress response in the central and autonomic nervous systems (27). Additionally, acute stressors induce brain activation, increasing the bioavailability of cortisol and catecholamine (28). Therefore, multiple mechanisms lead to myocardial damage and subtle ongoing cardiac dysfunction (29). Hypoconnectivity of central brain regions is associated with autonomic functions and regulation of the limbic system (28). Furthermore, this case showed the bilateral frontal lobe’s chronic small ischemic focus (Figure 2). Anxiety may be related to the prefrontal regions in the neuroimaging findings (30). As such, this may be the connection between the brain and heart.

Another theory suggests that some substances may be transmitted through the PFO. Migraine can be triggered by the passage of serotonin through the PFO, hence avoiding the metabolic transformation in the lungs and gaining entry to the systemic circulation at a higher concentration, causing ischemia, cortical irritability, depression, and/or migraine (31). Compared with healthy controls, children and adolescents with migraines are at higher risk of anxiety and depression symptoms (32). This indicates that anxiety, depression, and migraine may have similar mechanisms.

To our knowledge, such a shunting mechanism and theory have never been reported in patients with acute systemic hypoxemia, right-to-left shunting, severe anxiety, and depression. The cause and pathogenesis of anxiety and depression in PFO cases remain to be understood. This may be an implication for psychiatrists, specialists in psychosomatic medicine, internists, pulmonologists, and cardiologists. Furthermore, patent foramen ovale may profoundly affect the mechanism of anxiety and depression. It may present a new avenue for the exploration of the relationships between psychology and medicine. However, additional research is needed in the future to support these findings. We need to conduct larger studies involving a more diverse population to validate the findings and establish a stronger correlation between anxiety and PFO. Prospective studies and controlled trials are necessary to determine the underlying mechanisms and causality.

## Data availability statement

The original contributions presented in the study are included in the article/supplementary material, further inquiries can be directed to the corresponding author.

## Ethics statement

The studies involving humans were approved by Hebei General Hospital and complied strictly with ethical requirements. Ethics Review No. (2023) scientific ethics no. (59). The studies were conducted in accordance with the local legislation and institutional requirements. The participants provided their written informed consent to participate in this study. Written informed consent was obtained from the individual(s) for the publication of any potentially identifiable images or data included in this article. Written informed consent was obtained from the participant/patient(s) for the publication of this case report.

## Author contributions

XW and AN contributed to the conception and design of the study. RJ provided the images. XZ wrote the manuscript. All authors contributed to the manuscript revision, and read and approved the submitted version.

## Conflict of interest

The authors declare that the research was conducted in the absence of any commercial or financial relationships that could be construed as a potential conflict of interest.

## Publisher’s note

All claims expressed in this article are solely those of the authors and do not necessarily represent those of their affiliated organizations, or those of the publisher, the editors and the reviewers. Any product that may be evaluated in this article, or claim that may be made by its manufacturer, is not guaranteed or endorsed by the publisher.
